# Transcriptional changes in *Toxoplasma gondii* in response to treatment with monensin

**DOI:** 10.1186/s13071-020-3970-1

**Published:** 2020-02-18

**Authors:** Bintao Zhai, Jun-Jun He, Hany M. Elsheikha, Jie-Xi Li, Xing-Quan Zhu, Xiaoye Yang

**Affiliations:** 1 College of Veterinary Medicine, Inner Mongolia Agricultural University, Hohhot, 010018 Inner Mongolia Autonomous Region People’s Republic of China; 2 State Key Laboratory of Veterinary Etiological Biology, Key Laboratory of Veterinary Parasitology of Gansu Province, Lanzhou Veterinary Research Institute, Chinese Academy of Agricultural Sciences, Lanzhou, 730046 Gansu People’s Republic of China; 3 Faculty of Medicine and Health Sciences, School of Veterinary Medicine and Science, University of Nottingham, Sutton Bonington Campus, Loughborough, LE12 5RD UK; 4 Jiangsu Co-innovation Center for the Prevention and Control of Important Animal Infectious Diseases and Zoonoses, Yangzhou University College of Veterinary Medicine, Yangzhou, 225009 Jiangsu People’s Republic of China

**Keywords:** *Toxoplasma gondii*, Monensin, PK-15 cells, RNA-sequencing

## Abstract

**Background:**

Infection with the apicomplexan protozoan parasite *T. gondii* can cause severe and potentially fatal cerebral and ocular disease, especially in immunocompromised individuals. The anticoccidial ionophore drug monensin has been shown to have anti-*Toxoplasma gondii* properties. However, the comprehensive molecular mechanisms that underlie the effect of monensin on *T. gondii* are still largely unknown. We hypothesized that analysis of *T. gondii* transcriptional changes induced by monensin treatment can reveal new aspects of the mechanism of action of monensin against *T. gondii*.

**Methods:**

Porcine kidney (PK)-15 cells were infected with tachyzoites of *T. gondii* RH strain. Three hours post-infection, PK-15 cells were treated with 0.1 μM monensin, while control cells were treated with medium only. PK-15 cells containing intracellular tachyzoites were harvested at 6 and 24 h post-treatment, and the transcriptomic profiles of *T. gondii*-infected PK-15 cells were examined using high-throughput RNA sequencing (RNA-seq). Quantitative real-time PCR was used to verify the expression of 15 differentially expressed genes (DEGs) identified by RNA-seq analysis.

**Results:**

A total of 4868 downregulated genes and three upregulated genes were identified in monensin-treated *T. gondii*, indicating that most of *T. gondii* genes were suppressed by monensin. Kyoto Encyclopedia of Genes and Genomes (KEGG) pathway enrichment analysis of *T. gondii* DEGs showed that *T. gondii* metabolic and cellular pathways were significantly downregulated. Spliceosome, ribosome, and protein processing in endoplasmic reticulum were the top three most significantly enriched pathways out of the 30 highly enriched pathways detected in *T. gondii*. This result suggests that monensin, *via* down-regulation of protein biosynthesis in *T. gondii*, can limit the parasite growth and proliferation.

**Conclusions:**

Our findings provide a comprehensive insight into *T. gondii* genes and pathways with altered expression following monensin treatment. These data can be further explored to achieve better understanding of the specific mechanism of action of monensin against *T. gondii*.
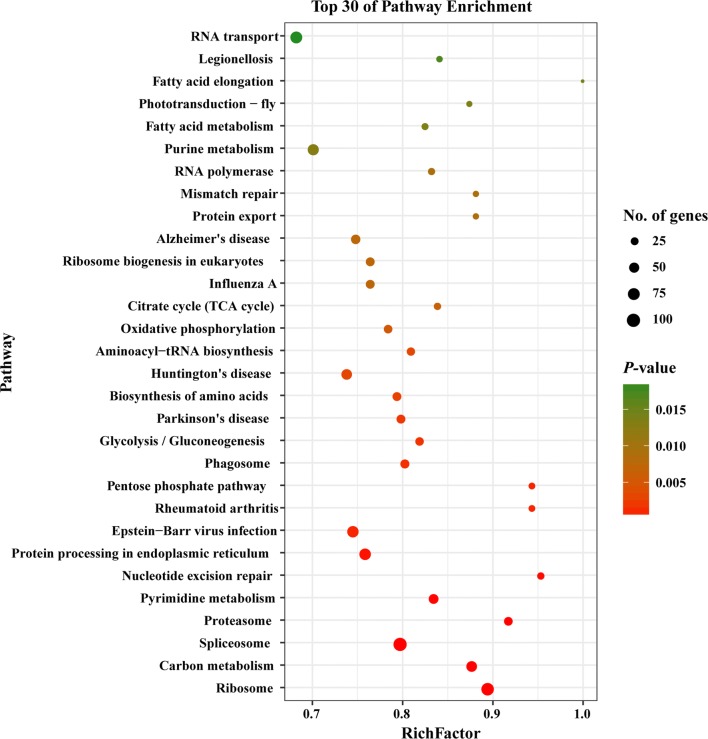

## Background

*Toxoplasma gondii* is one of the most successful opportunistic pathogens and has a wide range of intermediate hosts [1, 2]. This prolific parasite is estimated to cause latent infection in a third of the global human population [3]. While *T. gondii* is largely benign in immunocompetent individuals, infection with this parasite can cause severe inflammation of the retina, and in severely immunosuppressed patients, latent tissue cysts can reactivate in the brain causing life-threatening toxoplasmic encephalitis [4]. *Toxoplasma gondii* is also responsible for significant economic losses attributed to abortions of pregnant sheep following primary infection especially during early and mid-pregnancy [5].

In veterinary medicine, control of ovine toxoplasmosis relies on the use of decoquinate [6]. Also, monensin [7] and the folate inhibitor drugs, sulphamezathine and pyrimethamine [8], have been evaluated against *T. gondii* infection in pregnant sheep. There is a vaccine (Toxovax^®^, MSD Animal health) licensed for the prevention of abortion in sheep [9], although this vaccine suffers from a number of shortcomings [10]. Regarding humans, the first-line therapy for *T. gondii* infection is a combination of pyrimethamine and sulfadiazine. However, this regimen has some limitations because these drugs must be taken for a long duration, often cause side effects, and are incapable of eliminating the latent infection [11]. These drawbacks pose a major obstacle in conventional chemotherapy of toxoplasmosis in humans. To this end, efforts have been made to identify new and more effective medicines [12, 13] and to understand the mechanism of action [14] and perturbation associated with the currently used drugs [15].

One of the drugs that received more attention in recent years is monensin, which is an ionophore antibiotic used to treat coccidiosis in poultry and dairy animals. Monensin has shown antiparasitic activity against *T. gondii in vitro* [16, 17] and in sheep [7]. Through induction of oxidative stress, monensin disrupts the mitochondrial function, and induces an arrest of the cell cycle and autophagy-like cell death in *T. gondii* [14]. Given the promising anti-*T. gondii* activity of monensin, further understanding of its mechanism of action could reveal new targets for drug development against *T. gondii*. The transcriptomic profile of *T. gondii*-infected porcine kidney (PK-15) cells has been reported [18]. However, comprehensive understanding of how monensin treatment alters the transcriptome of *T. gondii* remains unknown.

In the present study, we profiled global gene expression in *T. gondii* following treatment of *T. gondii*-infected PK-15 cells with monensin using high-throughput RNA-sequencing (RNA-seq) analysis. Our data showed that monensin can cause genome-wide transcriptional changes in *T. gondii*.

## Methods

### *Toxoplasma gondii* culture

Tachyzoites of *T. gondii* RH strain were cultured and maintained in porcine (*Sus scrofa*) kidney (PK-15) cell monolayers. PK-15 cells were obtained from the American Tissue Culture Collection (ATCC^®^ CCL-33™; Maryland, USA) and cultured in Dulbecco’s Modified Eagleʼs Medium (DMEM, HyClone, Shanghai, China) supplemented with 10% fetal bovine serum (Gibco, Maryland, USA) at 37 °C in 5% CO_2_. Tachyzoites were harvested when 80% of the infected PK-15 cells had lysed. The infected cells and egressed tachyzoites were passed through a 22-gauge needle 20 times to rupture any remaining PK-15 cells. The supernatant was removed by centrifugation at 350×*g* for 10 min at 4 °C, and the tachyzoites were resuspended in 3 ml DMEM. The final purified tachyzoites were counted using a hemocytometer.

### Monensin treatment

PK-15 cells were infected with tachyzoites at a multiplicity of infection of 3 (3 tachyzoites: 1 PK-15 cell). Three hours post-infection, 12 T25 tissue culture flasks were randomly divided into four groups (3 flasks/group). The two treatment groups included M6 (*T. gondii*-infected cells at 6 h post-monensin treatment) and M24 (*T. gondii*-infected cells at 24 h post-monensin treatment). The two control groups (C6 and C24) were infected and untreated cells. The M6 and M24 groups were treated with monensin solution (Alfa Aesar, Ward Hill, USA) at a final concentration of 0.1 μM, while the control groups were treated with fresh medium without monensin. Each group included three biological replicates. The treated and control (untreated) cells were harvested at 6 and 24 h post-treatment and stored at -80 °C, until used for RNA extraction and RNA-seq.

### RNA extraction and RNA-seq analysis

Total RNA was individually extracted from each sample using TRIzol (Invitrogen China Ltd, Beijing, China) according to the manufacturer’s instructions. All extracted RNAs were treated with RNase-Free DNase (Ambion, Shanghai, China) to remove any residual genomic DNA. The integrity and quantity of all RNA samples were examined using the Agilent 2100 Bioanalyzer (Agilent Technologies, Santa Clara, CA, USA) and a NanoDrop^TM^ spectrophotometer (Thermo Scientific, Wilmington, DE, USA), respectively. Five micrograms of total RNA were used for the construction of the transcriptome libraries and 100-bp paired-end strand-specific RNA-sequencing was performed on the BGISEQ-500 Platform as per the manufacturer’s instructions.

### Sequence filtering, read mapping and analysis of differentially expressed genes (DEGs)

The raw sequencing data were processed using the FASTX tool (http://hannonlab.cshl.edu/fastx_toolkit/) to remove adaptor sequences, low-quality reads (quality value < 20), reads containing > 5% N rate and joint sequences before downstream analyses. StringTie [19] was used to reconstruct the transcripts guided by the genomic annotation information. Novel transcripts were identified using Cuffcompare (a tool of Cufflinks) [20]. The coding ability of new transcripts was predicted using Coding Potential Calculator [21]. The high-quality clean reads were then mapped to the reference genomes of pig (*Sus scrofa*) (ftp://ftp.ncbi.nlm.nih.gov/genomes/Sus_scrofa/) and *T. gondii* (ftp://ftp.ncbi.nlm.nih.gov/genomes/refseq/protozoa/Toxoplasma_gondii/latest_assembly_versions/GCF_000006565.2_TGA4) using HISAT and Bowtie 2 tools [22]. The gene expression level was calculated for each sample using the RSEM (RNA-seq by expectation-maximization) program [23] and the FPKM (fragments per kilobase of exon per million mapped fragments) method. DEseq2 software was used to identify the differentially expressed genes (DEGs). Gene expression with log2 fold change ≥ 1 or ≤ − 1, and adjusted *P*-value < 0.01 was considered as differentially expressed. Universal Protein Resource (UniProt) (https://www.uniprot.org/), Kyoto Encyclopedia of Genes and Genomes (KEGG) Orthology Based Annotation System 3.0 (KOBAS) (http://kobas.cbi.pku.edu.cn/index.php) and Gene Ontology (GO, http://geneontology.org/) were used for gene/protein functional annotation, pathway annotation and gene enrichment analyses, respectively. The GO enrichment analysis results were categorized according to the biological process (BP), cellular component (CC) and molecular function (MF). The RNA-seq, reads alignment and DEG identification were carried out at BGI-Shenzhen, China.

### Verification of RNA-seq results by qPCR

Quantitative real-time PCR (qPCR) was used to verify the RNA-seq results. The expression levels for 15 DEGs were determined by qPCR using the same RNA samples that were used for the sequencing. The RNA samples were reverse-transcribed to single strand cDNA using the PrimeScript^TM^ RT reagent Kit (TaKaRa, Dalian, China). Fifteen genes (nine host cell genes and six *T. gondii* genes) were randomly selected for qPCR verification and β-actin was used as the reference gene. All qPCR reactions were performed on the BIO-CFX96 system (Bio-Rad, California, USA) using SYBR Green GoTaq^®^ qPCR Master Mix (Promega, Beijing, China) following the manufacturer’s instructions. The primers used for qPCR are listed in the Additional file 1: Table S1. The selected genes were analyzed in triplicate. The qPCR cycling conditions included 95 °C for 2 min followed by 40 cycles of 95 °C for 10 s, 58 °C for 15 s, 72 °C for 40 s, and the temperatures of the melting curve analysis ranged from 72 to 95 °C. The 2^−ΔΔCq^ method was used to calculate the relative expression of each gene.

## Results

We analyzed the global gene expression of *T. gondii* infecting PK-15 cells in the absence or presence of 0.1 μM monensin treatment using an Illumina platform. The obtained sequences were aligned against pig and *T. gondii* genome sequences. More than 11.01 Gb of clean bases/reads were obtained from each treated and untreated sample (Additional file 2: Table S2).

### Differentially expressed genes (DEGs)

**Fig. 1:**
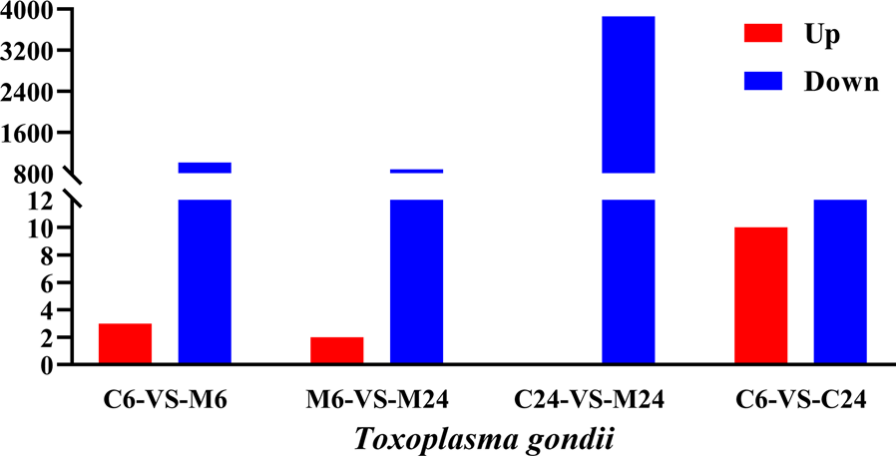
Distribution of the differently expressed genes (DEGs) of *T. gondii* across the examined groups. X-axis shows the difference between treated and untreated samples, and at two different time points (6 h and 24 h post-treatment). Y-axis represents the number of DEGs. Red and blue colours represent upregulated and downregulated DEGs, respectively

**Fig. 2:**
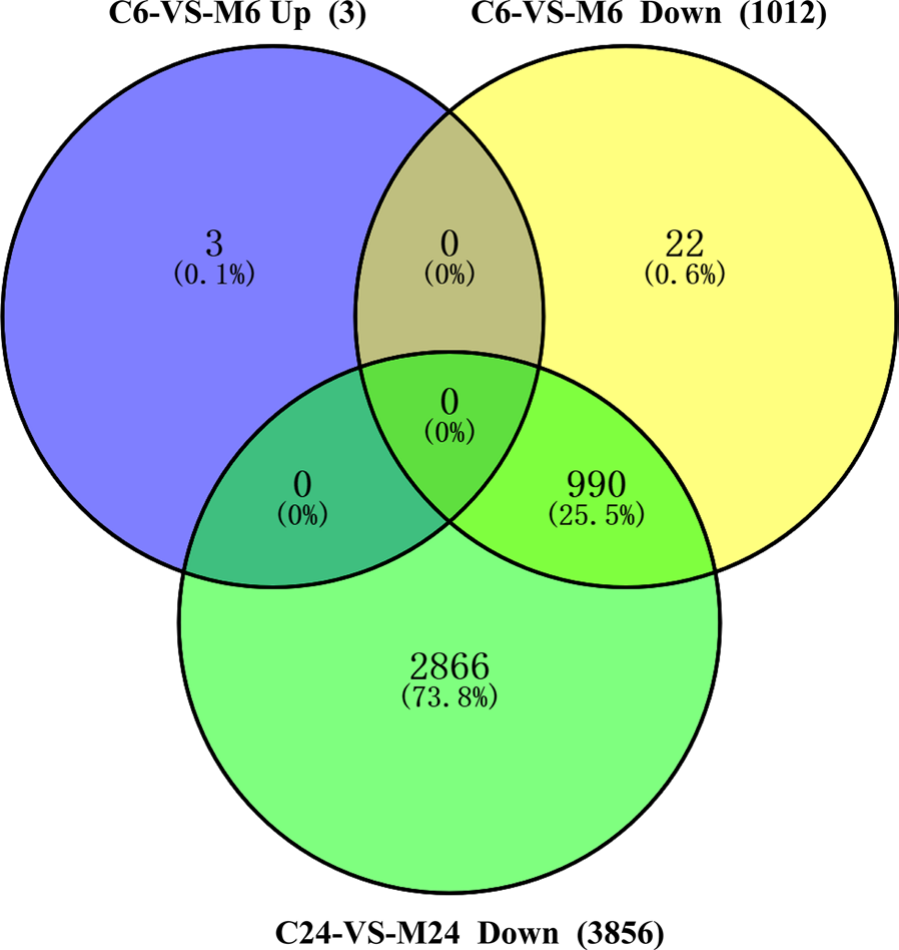
Venn diagram showing the overlap of the number of up and downregulated genes of *T. gondii* in C6 *vs* M6 group (6 h), and C24 *vs* M24 group (24 h)

**Fig. 3:**
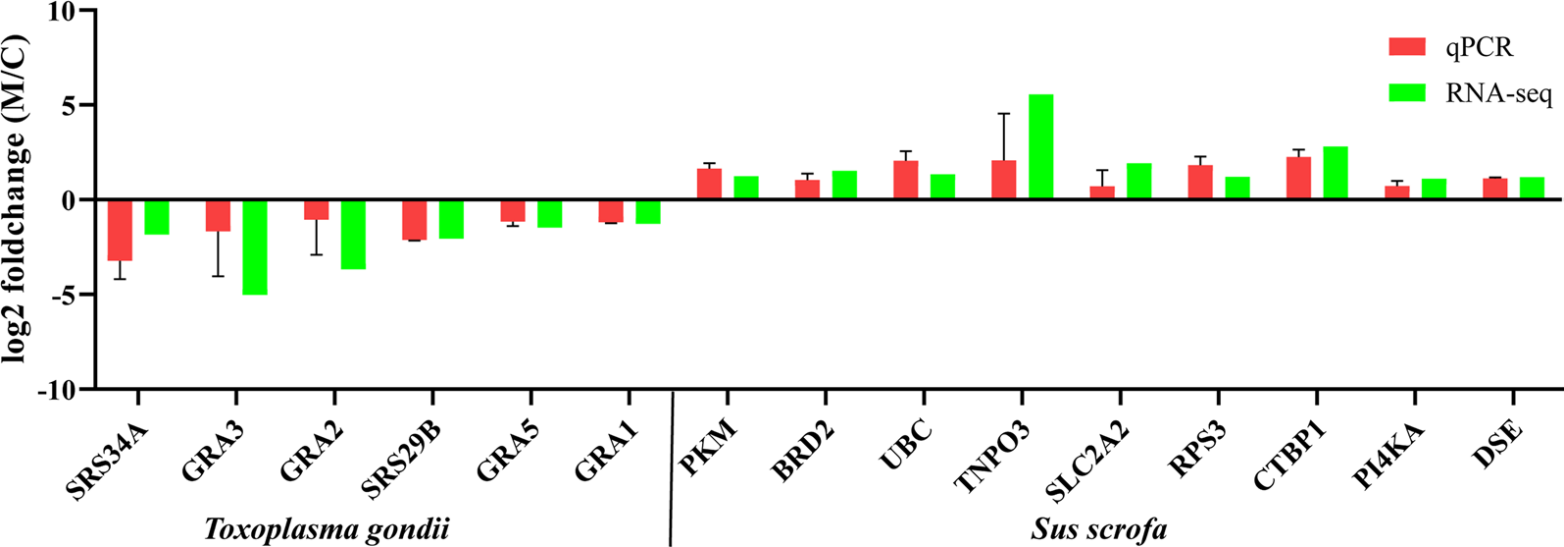
Verification of the RNA-seq data by using qPCR. Bars represent the mean fold changes of the expression of six *T. gondii* genes and nine porcine genes

Three upregulated and 1012 downregulated *T. gondii* genes were detected at 6 h post-treatment, while 3856 downregulated *T. gondii* genes were found at 24 h post-treatment (Fig. 1). Interestingly, 990 downregulated *T. gondii* DEGs were shared between monensin-treated samples at 6 and 24 h (Fig. 2). These 990 downregulated genes accounted for 97.8% of the downregulated genes at 6 h and 25.7% of the downregulated genes at 24 h post-treatment. The expression of 15 genes obtained through RNA-seq were confirmed by qPCR and the validation results are shown in Fig. 3.

### Gene Ontology (GO) analysis of the DEGs

**Fig. 4:**
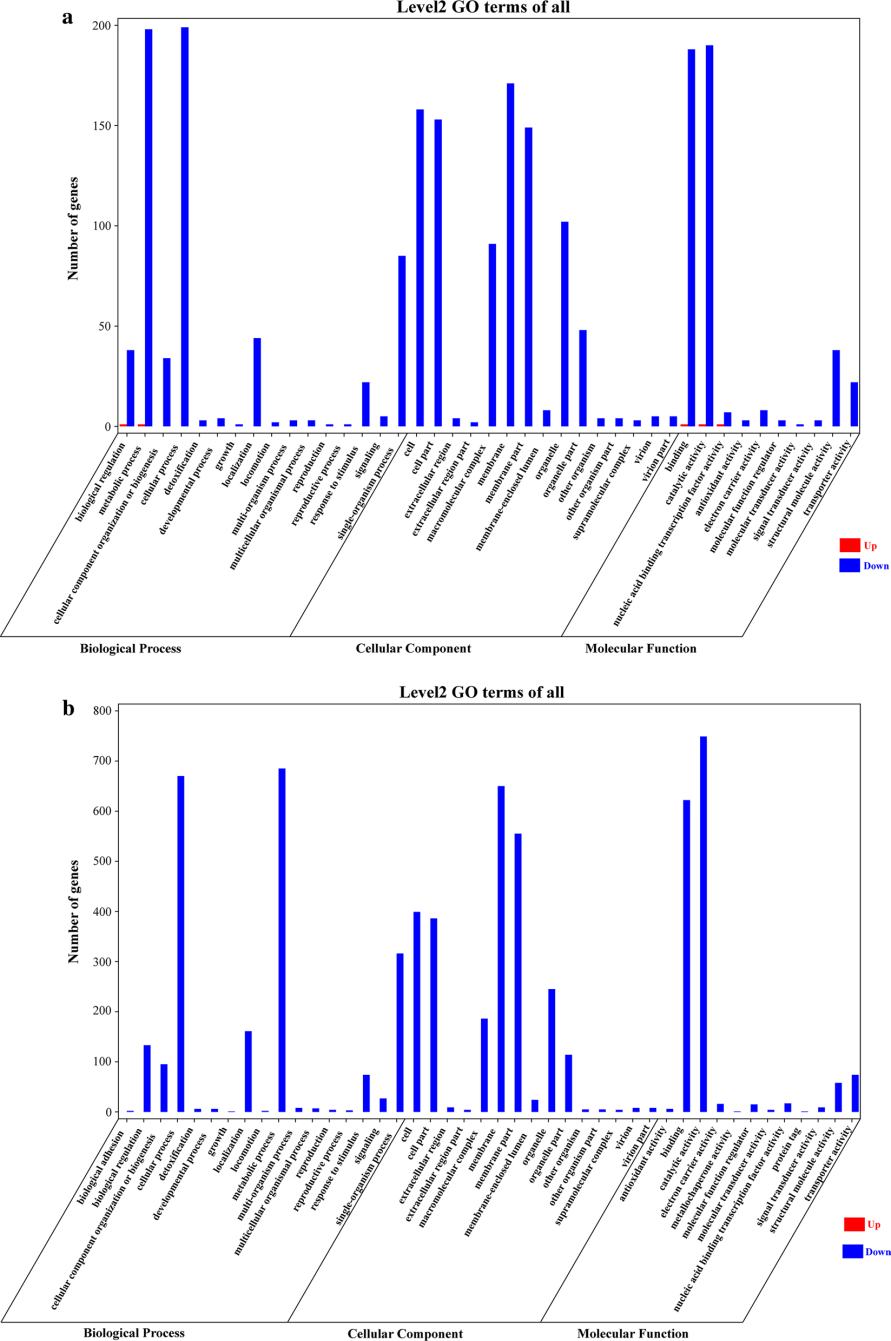
GO enrichment analysis of the differentially expressed genes (DEGs) of *T. gondii*. The bar graphs show the number of *T. gondii* DEGs enriched in GO terms belonging to the three GO categories, biological process, cellular component and molecular function, at 6 h (**a**) and 24 h (**b**). X-axis represents the GO terms and Y-axis represents the number of upregulated (Up) and downregulated (Down) genes in different GO terms

A total of 44 GO terms, including 17 biological process (BP) terms, 15 cellular component (CC) terms and 12 molecular function (MF) terms were significantly enriched for 4871 *T. gondii* DEGs (Fig. 4). Among the BP category at 6 and 24 h, the top two enriched GO terms were metabolic process and cellular process. In the CC category at 6 h, membrane and cell were the top two GO terms (Fig. 4a), while membrane and membrane part were the top two GO terms at 24 h (Fig. 4b). In the MF category at 6 and 24 h, the top two GO terms were catalytic activity and binding.

### KEGG pathway analysis

**Fig. 5:**
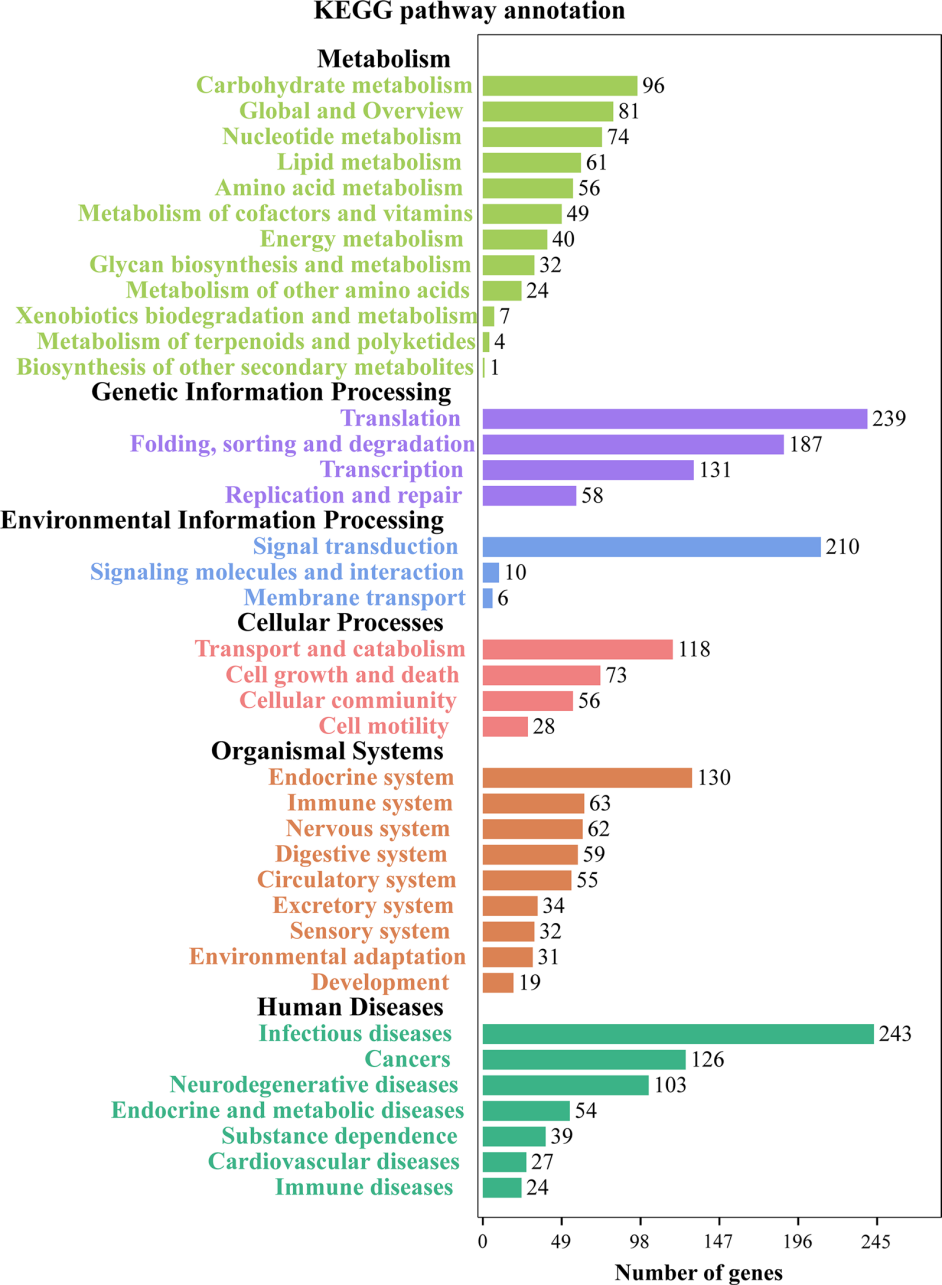
KEGG annotation of the DEGs in the transcriptome of *T. gondii*. X-axis label represents the number of DEGs in the corresponding KEGG pathways in each KEGG subsystem. Y-axis label represents main clusters of the KEGG pathways

**Fig. 6:**
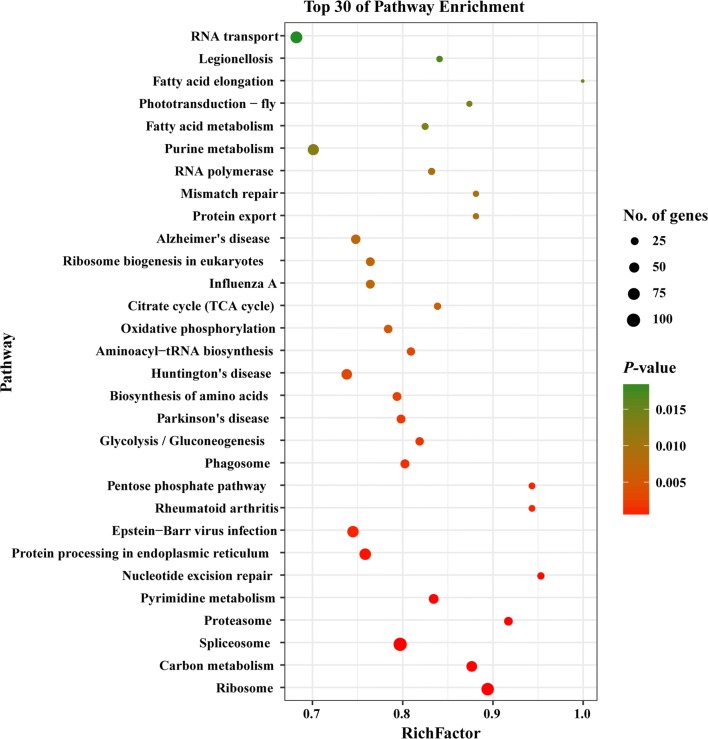
Scatterplot of the top 30 most enriched KEGG pathways of *T. gondii*. Y-axis label represents the distinct KEGG pathways, and X-axis label represents the Rich Factor. Rich Factor refers to the ratio of DEGs annotated in the pathway to total number of genes annotated in the pathway. The greater the Rich Factor, the greater the degree of pathway enrichment. Dot size represents the number of DEGs (bigger dots denote large DEG number and *vice versa*). The colours of the dots represent the *P*-values of enrichment. Red colour indicates high enrichment, while green colour indicates low enrichment

We also mapped the DEGs to six different KEGG subsystems, including metabolism, genetic information processing, environmental information processing, cellular processes, organismal systems and human diseases (Fig. 5). KEGG pathway analysis also showed that most of *T. gondii* DEGs were enriched in infectious diseases, signal transduction and translation. The 30 most significantly enriched pathways are shown in Fig. 6; spliceosome, ribosome, and protein processing in endoplasmic reticulum are the top three significantly enriched pathways in *T. gondii* (Additional file 3: Figure S1, Additional file 4: Figure S2, Additional file 5: Figure S3).

### Transcription factors (TFs) of DEGs

**Fig. 7:**
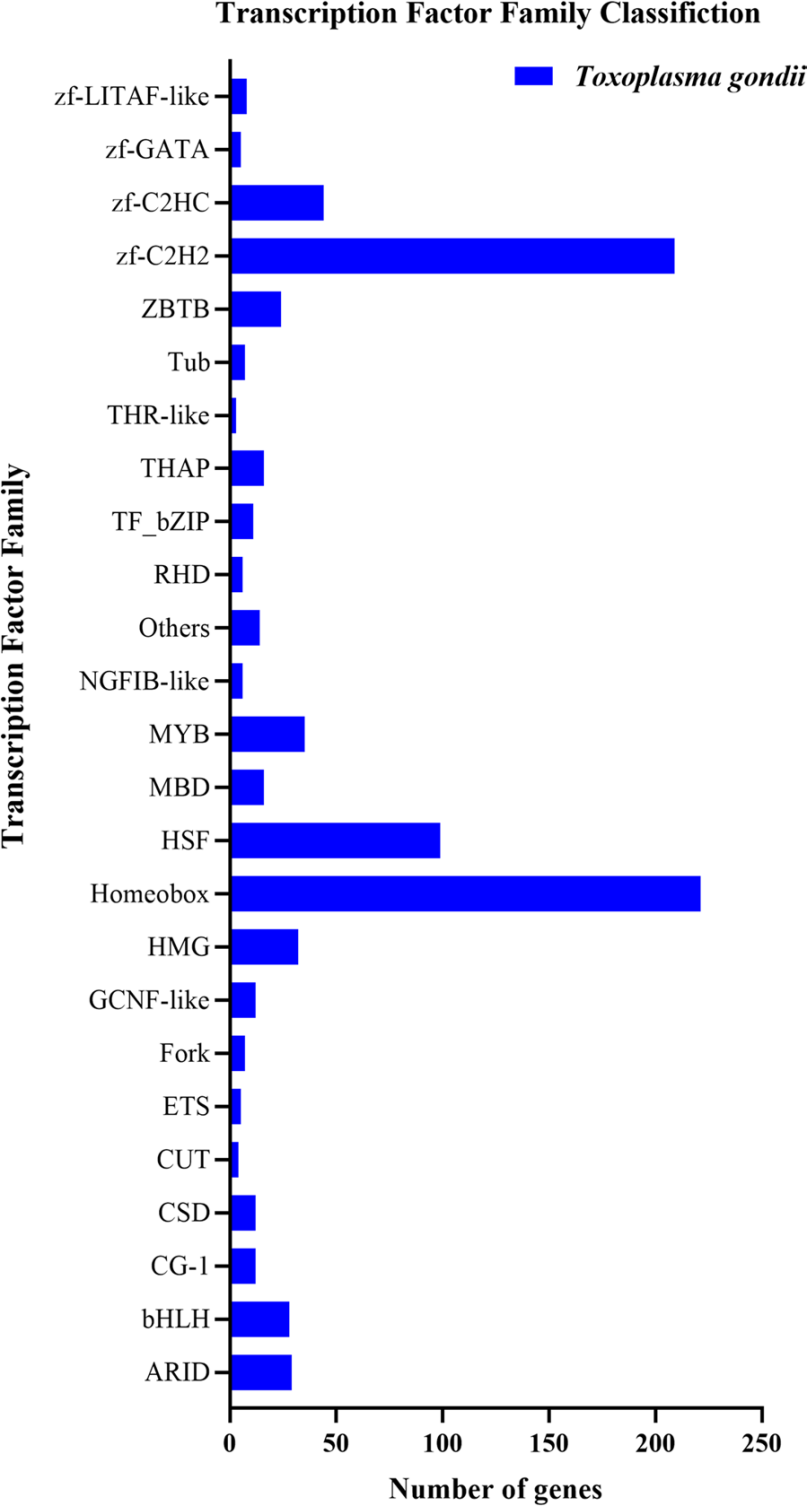
Classification of the differentially expressed TFs. The X-axis label represents the number of genes and the Y-axis label represents the transcription factor family names

TFs are key regulators of gene expression [24]. They bind to specific DNA sequences and activate or repress gene expression by DNA-binding domains (DBDs) [25]. Based on their DBDs, TFs can be classified into different families [26]. In our study, differentially expressed TFs were classified into 25 families (Fig. 7), and homeobox and zf-C2H2 were the two most significantly enriched TFs in *T. gondii*.

### Protein–protein interaction (PPI) of DEGs

**Fig. 8:**
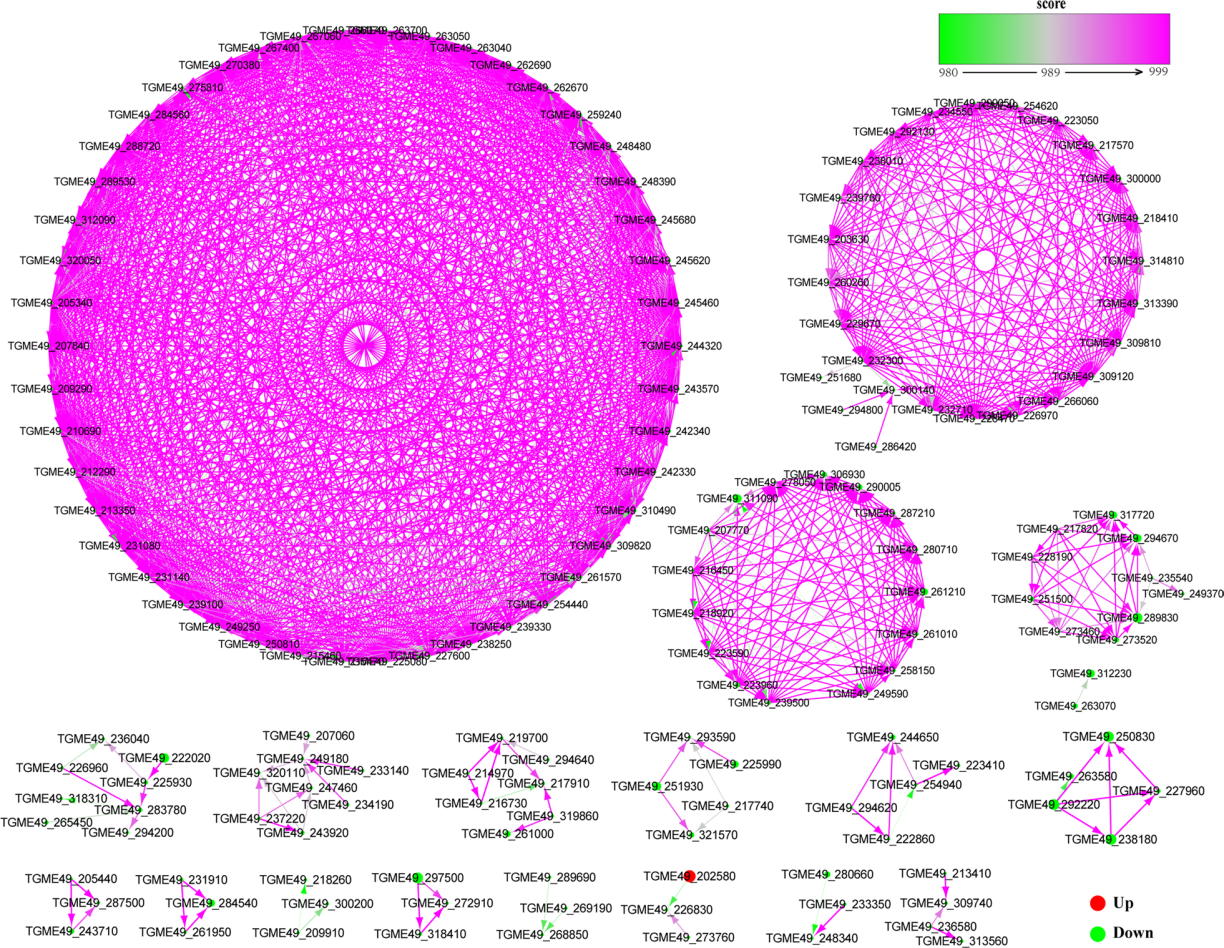
Transcriptional regulatory network analysis of *Toxoplasma gondii*. Protein–protein interaction (PPI) networks of DEGs of *T. gondii* at 6 h. The red and green dots denote the upregulated and downregulated genes, respectively

Using the String database prediction, PPI networks of *T. gondii* with a combined score > 980 at 6 h post-monensin treatment are shown in Fig. 8. TGME49_002580 (XM_018779214.1), which encodes ATPases associated with diverse cellular activities (AAA proteins), was the most enriched upregulated gene in *T. gondii*. Four proteins, TGME49_238180 (XM_018780522.1, K03037), TGME49_292220 (XM_002368522.2, K03033), TGME49_250830 (XM_018780938.1, K03031) and TGME49_227960 (XM_002366378.2, K03036), formed a separate mutual network. TGME49_238180, TGME49_292220 and TGME49_227960 regulate TGME49_250830, while TGME49_292220 regulates TGME49_250830, TGME49_227960 and TGME49_238180. These proteins (TGME49_292220 (K03033, Rpn3), TGME49_238180 (K030037, Rpn7), TGME49_227960 (K03036, Rpn6), and TGME49_250830 (K03031, Rpn12)) are the components of the 19S regulatory particle in the proteasome pathway (map03050, Additional file 6: Figure S4). The protein TGME49_289830 (XM_002368367.1, K03246) regulates two proteins, TGME49_294670 (XM_002370195.2, K03248) and TGME49_317720 (XM_018782917.1, K03251); where they all belong to the family of translation initiation factors (eIF3) of the RNA transport pathway. Additional file 7: Figure S5 shows *T. gondii* PPIs at 24 h where TGME49_210790 (XM_002371193.2), TGME49_266460 (XM_002368694.2), TGME49_297140 (XM_018782303.1), TGME49_275750 (XM_002371561.2) and TGME49_305010 (XM_002370254.1) are some of the proteins that warrant further studies.

## Discussion

The search for new anti*-Toxoplasma gondii* drugs has been active for several decades [12, 13], but only a few drugs are currently approved for use in humans [1, 27]. Although sulfa drugs can be effectively used for the prevention and control of *T. gondii* infection in humans and animals, their side effects should not be ignored [28]. Compared to the mainstream anti-*Toxoplasma* drugs (sulfa and ethylamines, trimethoprim combined with sulfamethoxazole), monensin seems to be less cytotoxic [29, 30]. The anticoccidial drug monensin has been shown to inhibit the viability and even damage the bradyzoite stage of *T. gondii* [29] and to prevent the shedding of oocysts from cats [31]. Monensin can also induce cell cycle arrest and autophagy, leading to death of *T. gondii* tachyzoites [32, 33], probably mediated by an oxidative stress-related mechanism [14]. Despite this body of literature describing the mechanisms that mediate the inhibitory effects of monensin against different life-cycle forms of *T. gondii*, the comprehensive mechanisms responsible for killing of *T. gondii* by monensin remains incompletely defined.

In this study, we used RNA-seq technology to identify the global transcriptomic changes in *T. gondii* caused by monensin treatment. We found 4868 downregulated genes and three upregulated genes in *T. gondii* following monensin treatment. The significant number of downregulated genes shows the overwhelming impact of monensin treatment on *T. gondii*, especially at 24 h post-treatment. We also performed GO enrichment analysis to analyze the significantly altered biological processes in *T. gondii* caused by monensin treatment. The two most significantly enriched BP GO terms at 6 and 24 h were metabolic process and cellular process. In the MF category, the top two GO terms were catalytic activity and binding at 6 and 24 h. For the CC category, membrane and membrane parts were the two most enriched GO terms at both 6 and 24 h (Fig. 4); these included membrane components that contribute to material transportation, membrane integration, environmental resistance and various biological functions that are essential for cell survival. These findings indicate that anti-*T. gondii* effects of monensin could be mediated by impairment of most of *T. gondii* biological processes and membrane components.

KEGG pathway analysis showed that spliceosome, ribosome and protein processing in the endoplasmic reticulum were the top three of the 30 most significantly enriched pathways in *T. gondii* (Fig. 6). Protein processing in the endoplasmic reticulum is a pathway that influences protein folding in the endoplasmic reticulum [34]. Proteolytic cleavage of effectors in the endoplasmic reticulum pathway is essential for the survival of *T. gondii* [35]. Additional file 5: Figure S3 shows that most genes involved in protein processing in the endoplasmic reticulum pathway are downregulated. Thus, we infer that monensin could suppress protein processing in the endoplasmic reticulum pathway in *T. gondii*, which would contribute to its anti-*T. gondii* activity.

The spliceosomes are RNA-protein complexes responsible for removal of introns (non-coding segments) from pre-messenger RNAs to form mature mRNAs in a process known as splicing [36]. Spliceosome components have been identified in *T. gondii* [37]. Our analysis showed that all DEGs involved in the spliceosome pathway are downregulated by monensin (Additional file 3: Figure S1). Ribosome biogenesis is closely related to multiple cellular signaling pathways and any defects in ribosome production can cause many diseases, and even death [38]. The ribosome profiling at the level of transcription and translation of *T. gondii* has been reported [39]. However, how the ribosome of *T. gondii* is altered by monensin remains unknown. Our analysis showed that DEGs involved in ribosome biogenesis are significantly downregulated by monensin (Additional file 4: Figure S2). These findings indicate that monensin can also interfere with genes involved in mRNA translation and ribosome biogenesis, which can restrict the growth of *T. gondii*.

The biogenesis of the spliceosome and ribosome are regulated by transcription factors (TFs). We found that homeobox and zf-C2H2 were the two most significantly enriched TFs (Fig. 7). The homeobox TF regulates the expression of genes associated with various developmental processes in animals, fungi and plants [40]. The zf-C2H2 TF family contains a small protein structural motif, the zinc finger (zf), which coordinates one or more zinc ions (Zn_2_^+^) [41]. TFs containing zinc fingers have been implicated in a variety of biological processes in *T. gondii* [42, 43]. For example, depletion of TgZNF2 in *T. gondii* caused an arrest of the parasite growth at the G1 phase of the cell cycle and accumulation of poly(A) RNA in their nucleus [43]. Thus, monensin-induced downregulation of these two TFs, homeobox and zf-C2H2, may disrupt the growth and development of *T. gondii*, further elucidating more aspects of monensin mode of action against *T. gondii*.

The PPI analysis revealed several proteins that were downregulated by monensin, including TGME49_210790, TGME49_305010, TGME49_266460 and TGME49_002580. TGME49_002580 is ATPase, AAA family protein, which plays critical roles in various cellular processes [44]. TGME49_210790 (XM_002371193.2) encodes a putative dihydroorotate dehydrogenase (DHODH), which mediates the fourth step of *de novo* pyrimidine biosynthesis [45]. In *T. gondii*, disruption of *de novo* pyrimidine synthesis results in uracil auxotrophy, virulence attenuation and inability to establish latent infection [46]. Inhibition of the activity of *T. gondii* dihydroorotate dehydrogenase (TgDHODH) can potentiate the growth inhibiting potential of 1-hydroxyquinolones in *T. gondii* [45]. TGME49_305010 (XM_002370254.1) is putatively encoded as pre-mRNA branch site protein p14, which is associated with U2 small nuclear ribonucleoprotein particles (snRNPs) and participates in the spliceosome (map03040) pathway. TGME49_266460 (XM_002368694.2) encodes a small ubiquitin-like family modifier (SUMO) belonging to the Ubl family, while only one gene is encoded by SUMO in lower eukaryotes, including *T. gondii* [47]. A previous study of *T. gondii* SUMO proteomics revealed over 100 sumoylated proteins involved in translation, metabolism, post-translational modification, and protein degradation [48]. Altering these proteins in *T. gondii* may be lethal, which would then contribute to the anti-*T. gondii* activity of monensin.

## Conclusions

This study examined the transcriptomic landscape of *T. gondii* infecting PK-15 cells treated with monensin and identified monensin-induced DEGs in *T. gondii*. Our genome-wide transcriptional analysis revealed that 4868 *T. gondii* genes were downregulated in treated cell cultures, suggesting that monensin can suppress the expression of the majority of *T. gondii* genes. Also, monensin treatment appears to adversely influence various crucial metabolic and cellular processes of *T. gondii*, such as spliceosome, ribosome and protein processing in the endoplasmic reticulum. Additionally, monensin induced downregulation of two transcription factors, homeobox and zf-C2H2, in *T. gondii*. Further analysis of the identified transcriptional changes can provide useful information for better understanding of the mechanism of action of monensin against *T. gondii*.

## Supplementary information

**Additional file 1: Table S1.** Primer sequences used for qPCR analysis.

**Additional file 2: Table S2.** Quality metrics of the clean reads.

**Additional file 3: Figure S1.** The spliceosome pathway.

**Additional file 4: Figure S2.** The ribosome pathway.

**Additional file 5: Figure S3.** Protein processing in the endoplasmic reticulum pathway.

**Additional file 6: Figure S4.** Proteasome pathway (map03050). The proteasome is a protein-destroying apparatus involved in many essential cellular functions. The green box Rpn3 represents TGME49_292220 (K03033), Rpn7 represents TGME49_238180 (K030037), Rpn6 represents TGME49_227960 (K03036), and Rpn12 represents TGME49_250830 (K03031).

**Additional file 7: Figure S5.** *Toxoplasma gondii* PPIs at 24 h post-treatment.

## Data Availability

The RNA-seq data obtained in this study were deposited in the National Center for Biotechnology Information (NCBI) Sequence Read Archive (SRA) database (https://www.ncbi.nlm.nih.gov/sra) under accession number SUB6209220.

## Abbreviations

qPCR: Quantitative real-time PCR
DEGs: Differentially expressed genes
TE: Toxoplasmic encephalitis
CNS: Central nervous system
PK-15: Porcine kidney-15
ATCC: American Tissue Culture Collection
DMEM: Dulbecco’s Modified Eagleʼs Medium
MOI: Multiplicity of infection
KEGG: Kyoto Encyclopedia of Genes and Genomes
GO: Gene Ontology
BP: Biological process
CC: Cellular component
MF: Molecular function
RNA-seq: RNA-sequencing
FPKM: Fragments per kilobase of exon per million mapped fragments
TFs: Transcription factors
DBD: DNA-binding domain
PPI: Protein–protein interactions
zf: Zinc finger
C2H2: Cys2His2-like fold group
