# Supplementary material for: Transcriptional changes in Toxoplasma gondii in response to treatment with monensin
Source: Parasit Vectors. 2020 Feb 18;13:84. doi: 10.1186/s13071-020-3970-1 (PMC7029487; doi:10.1186/s13071-020-3970-1)
Supplement: Supplementary file 1 — Additional file 1: Table S1. Primer sequences used for qPCR analysis. [file 13071_2020_3970_MOESM1_ESM.doc]

**Additional File 1: Table S1 Primer sequences used for qPCR analysis.**

| **Gene** | **Forward primer** | **Reverse primer** | **NCBI accession number** |
| --- | --- | --- | --- |
| 1. ***gondii*** |  |  |  |
| SRS34A | GAAGTTGGTGGTAACGGGAATA | GAGTCACTTCGGAGAACAGAAC | XM_018781602.1 |
| GRA3 | ACAGGAGTGCGGGATACAAC | TGCGTTCTTTTCACTGATGC | XM_002366330.2 |
| GRA2 | TAAGTGCGAGCAACATCTCTAC | TGACCCTTTCTGTCATCAGTTC | XM_002366354.2 |
| SRS29B | GCCTCATCGGTCGTCAATAA | GTCATTGTAGTGGGTCCTTCC | XM_002368164.2 |
| GRA5 | GCAAGAAAGGTGCCGAAATG | GATCTGAGGTTTCTCCGCTAAC | XM_002369230.2 |
| GRA1 | GCTGTGGAAAGTTGATGGTTG | CTGAGAAAGGACTGGAGAACTG | XM_002365660.2 |
| **PK-15 cells** |  |  |  |
| PKM | CCTGTGGCTGGACTATAAGAAC | CTGCTTCACCAGCAAAGAAATAA | XM_021099123.1 |
| BRD2 | GCGTCTTTTGCCTCTTTCAC | TGGCTTATTTCCCTGCATTC | XM_021097901.1 |
| UBC | CGTTCCCAATCTCTCTGTGG | GGATGCCCTCTTTTTCTTGA | XM_021074018.1 |
| TNPO3 | GCCTCTTTACGGGACTCATTAC | GTAGAGCAAGGTCGGCTATTG | XM_021079117.1 |
| SLC2A2 | CAGATCATAGGCCTCGACTTTC | CTGGACAGAAGAAGAGCATCAG | NM_001097417.1 |
| RPS3 | GAGGGCAGTGTAGAGCTTTATG | CGAGGAGTTTGTATCGCAGAG | NM_001044601.1 |
| CTBP1 | TCCTTCGCGTCCTTGTTAAG | CCGACAGCTAAGCAAACAAATC | XM_021100872.1 |
| PI4KA | ACTCGGCCACCAAAGATTAC | CAGGTGCTCATCCTGGATATTG | XM_001929569.6 |
| DSE | GTCCCTGAAGTGAAGGACTATG | CGCTCAGCTGTCTTCCTAAA | XM_021091551.1 |

*Abbreviation*: qPCR, Quantitative real-time PCR; PK-15, porcine kidney cells; *T. gondii*, *Toxoplasma gondii*.
