# Supplementary material for: Transcriptional changes in Toxoplasma gondii in response to treatment with monensin
Source: Parasit Vectors. 2020 Feb 18;13:84. doi: 10.1186/s13071-020-3970-1 (PMC7029487; doi:10.1186/s13071-020-3970-1)
Supplement: Supplementary file 2 — Additional file 2: Table S2. Quality metrics of the clean reads. [file 13071_2020_3970_MOESM2_ESM.doc]

**Additional File2: Table S2** **Quality metrics of the clean reads**

| Sample | Total raw reads (Mb) | Total clean reads (Mb) | Total clean bases (Gb) | Clean reads  Q20 (%) | Clean reads Q30 (%) | Clean reads ratio (%) |
| --- | --- | --- | --- | --- | --- | --- |
| C24_1 | 127.00 | 109.35 | 10.94 | 96.00 | 87.19 | 86.10 |
| C24_2 | 127.00 | 110.94 | 11.09 | 96.20 | 87.75 | 87.35 |
| C24_3 | 127.00 | 109.76 | 10.98 | 95.90 | 87.02 | 86.42 |
| C6_1 | 129.49 | 110.16 | 11.02 | 95.97 | 87.10 | 85.07 |
| C6_2 | 124.51 | 110.15 | 11.01 | 96.38 | 87.95 | 88.46 |
| C6_3 | 124.51 | 110.05 | 11.00 | 96.24 | 87.83 | 88.38 |
| M24_1 | 127.00 | 110.08 | 11.01 | 96.05 | 87.31 | 86.68 |
| M24_2 | 122.02 | 109.91 | 10.99 | 96.61 | 88.81 | 90.07 |
| M24_3 | 124.51 | 109.74 | 10.97 | 96.29 | 88.09 | 88.14 |
| M6_1 | 129.49 | 111.07 | 11.11 | 95.94 | 87.02 | 85.78 |
| M6_2 | 127.00 | 110.07 | 11.01 | 96.06 | 87.37 | 86.67 |
| M6_3 | 124.51 | 110.10 | 11.01 | 96.50 | 88.29 | 88.43 |

Samples: Sample group names, C: Control group, M: Drug treatment group

Total Raw Reads (Mb): The reads number before filtering, Unit: Mb

Total Clean Reads/Bases (Mb/Gb): The reads/bases number after filtering, Unit: Mb/Gb

Clean Reads Q20/30 (%): The Q20/30 value for the clean reads

Clean Reads Ratio (%): The ratio of the number of clean reads
