# Supplementary figures and images for: Transcriptional changes in Toxoplasma gondii in response to treatment with monensin
Source: Parasit Vectors. 2020 Feb 18;13:84. doi: 10.1186/s13071-020-3970-1 (PMC7029487; doi:10.1186/s13071-020-3970-1)

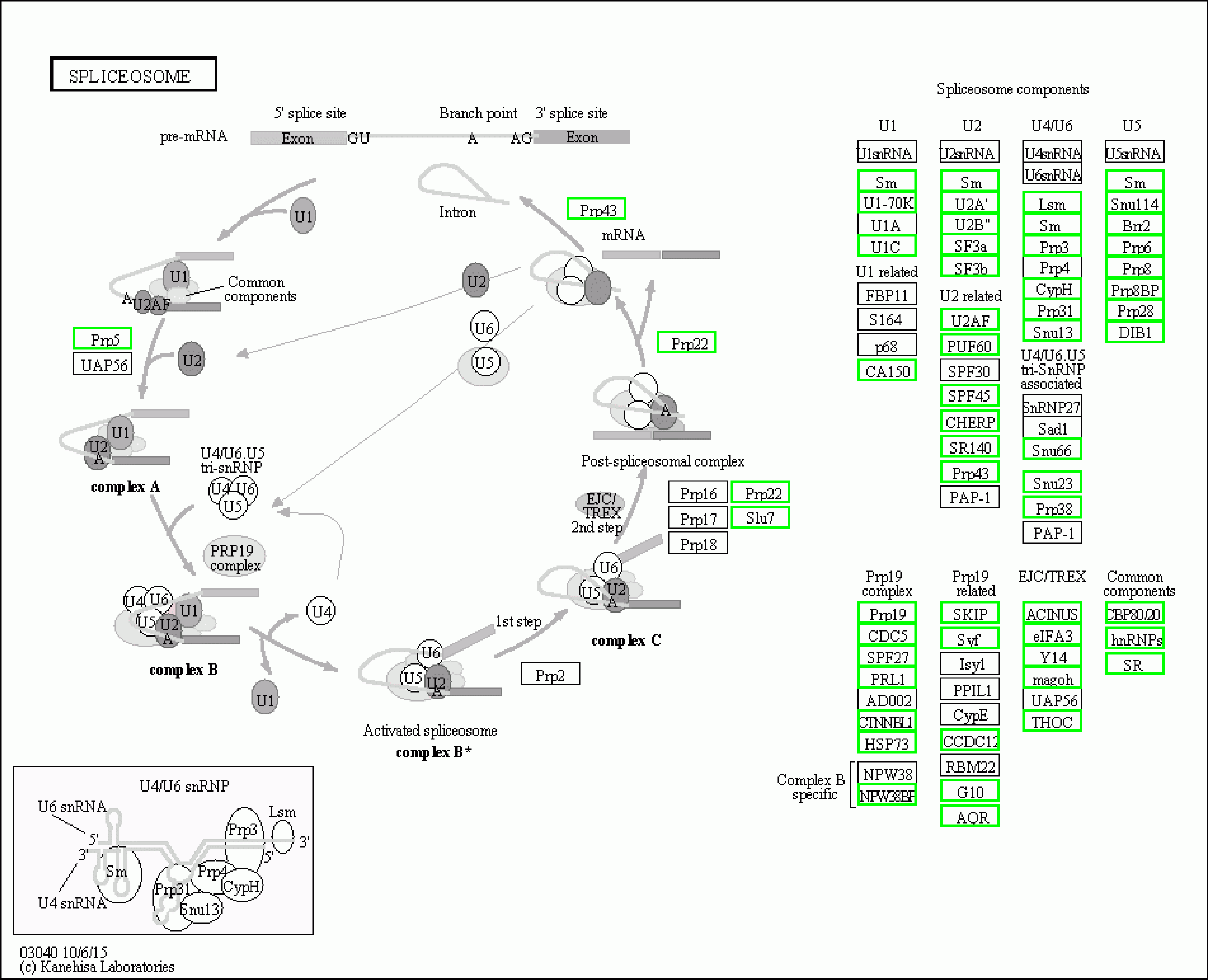

Supplement: Supplementary file 3 — Additional file 3: Figure S1. The spliceosome pathway. [file 13071_2020_3970_MOESM3_ESM.tif]

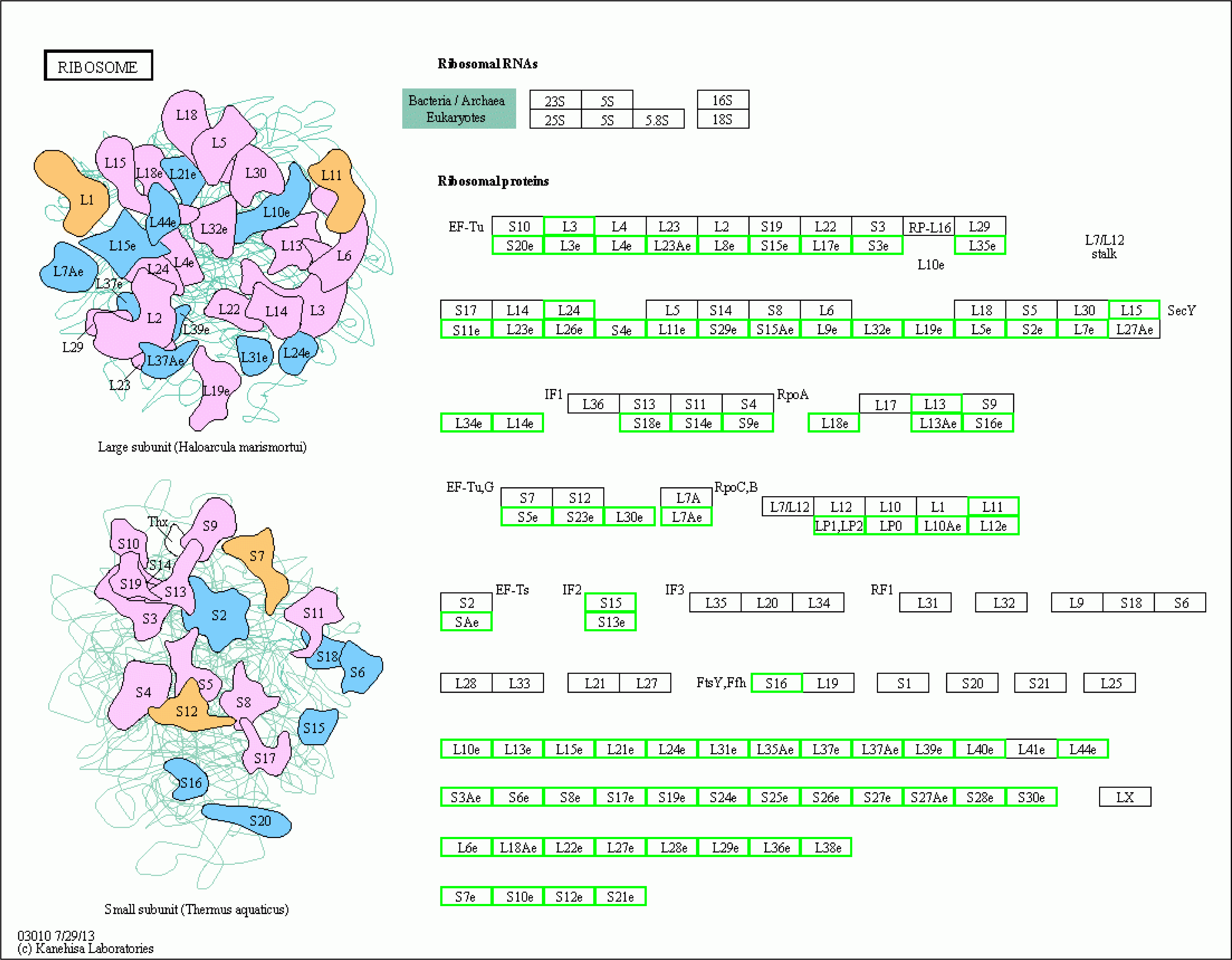

Supplement: Supplementary file 4 — Additional file 4: Figure S2. The ribosome pathway. [file 13071_2020_3970_MOESM4_ESM.tif]

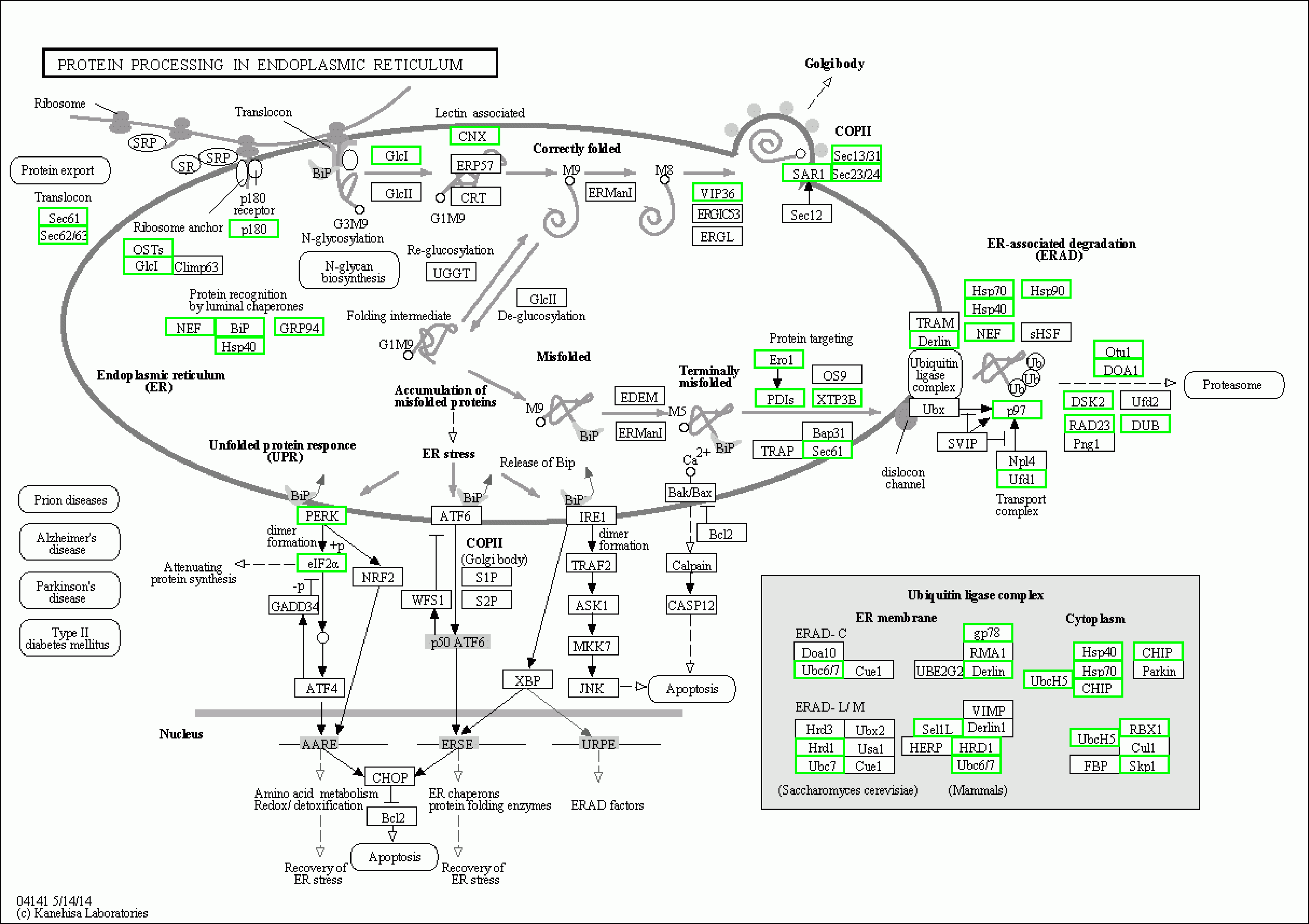

Supplement: Supplementary file 5 — Additional file 5: Figure S3. Protein processing in the endoplasmic reticulum pathway. [file 13071_2020_3970_MOESM5_ESM.tif]

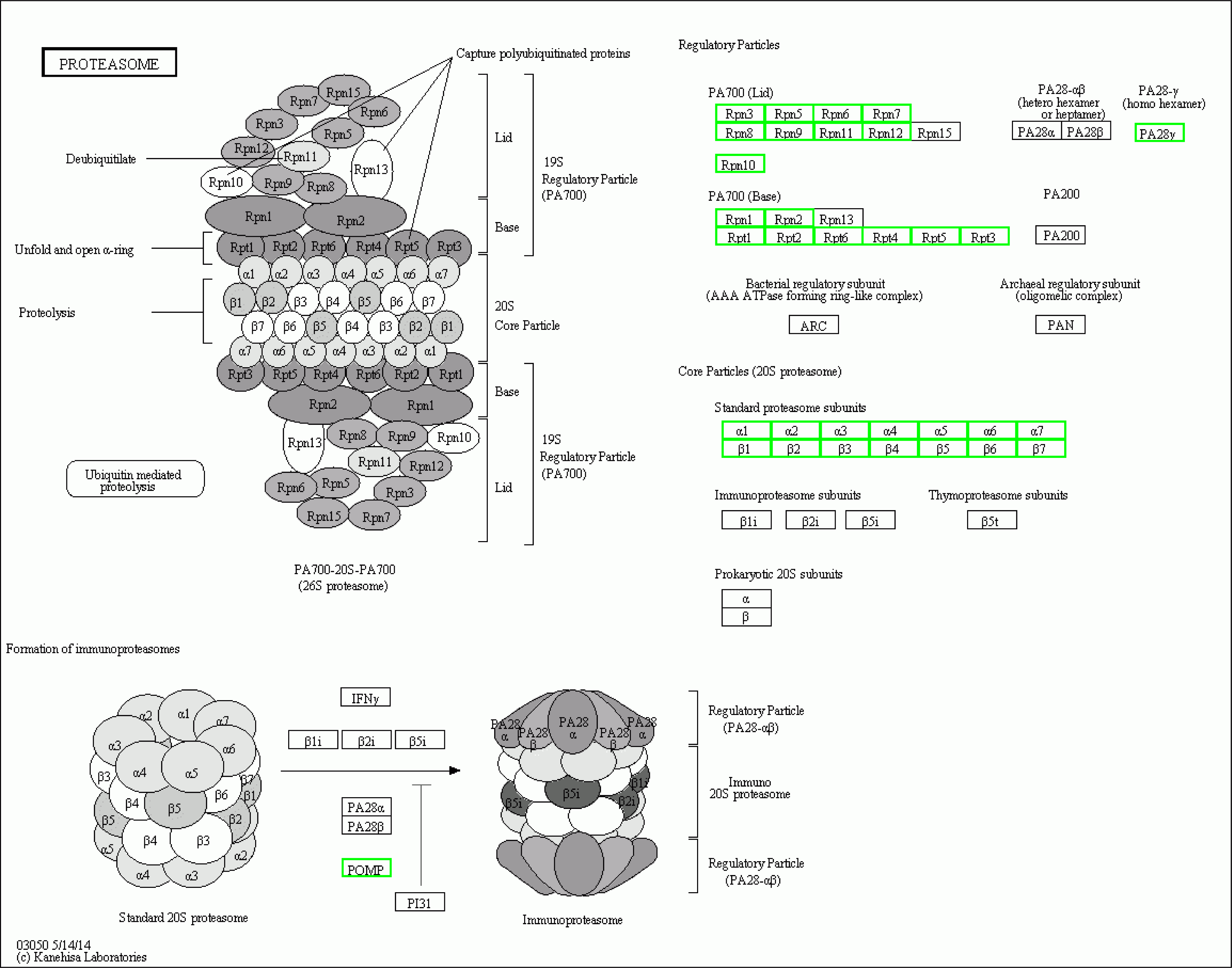

Supplement: Supplementary file 6 — Additional file 6: Figure S4. Proteasome pathway (map03050). The proteasome is a protein-destroying apparatus involved in many essential cellular functions. The green box Rpn3 represents TGME49_292220 (K03033), Rpn7 represents TGME49_238180 (K030037), Rpn6 represents TGME49_227960 (K03036), and Rpn12 represents TGME49_250830 (K03031). [file 13071_2020_3970_MOESM6_ESM.tif]

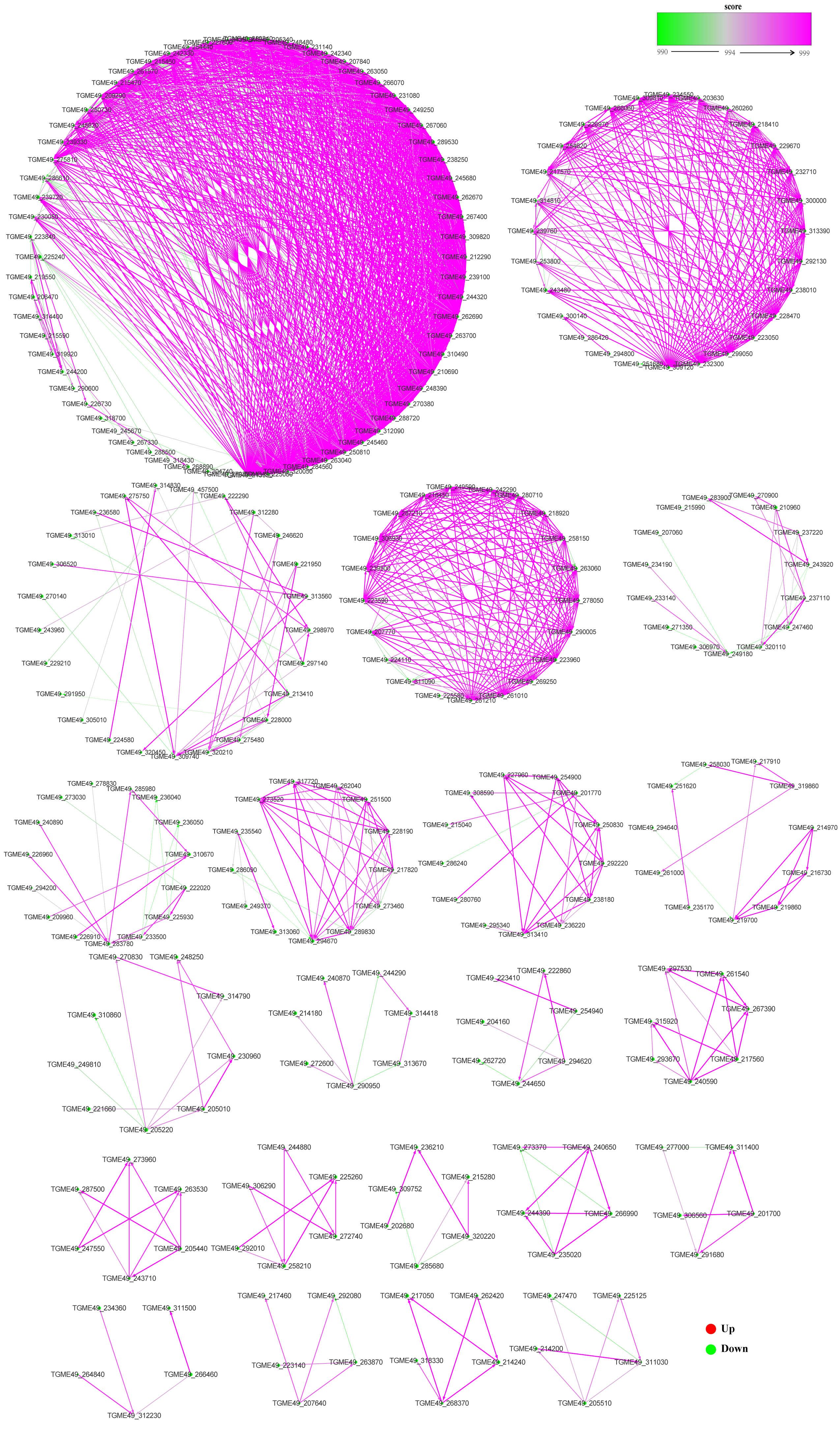

Supplement: Supplementary file 7 — Additional file 7: Figure S5. Toxoplasma gondii PPIs at 24 h post-treatment. [file 13071_2020_3970_MOESM7_ESM.tif]
